# Correlation of Optic Nerve Sheath Diameter With Severity and Outcome in Head Injury: Ultrasonographic and CT Evaluation

**DOI:** 10.7759/cureus.99126

**Published:** 2025-12-13

**Authors:** Syed Ali Mehsam, Sarosh Alam, Zunaira Rizwan, Haris Hanif, Fatima Tariq, Saharish Mansoor Khan

**Affiliations:** 1 Department of General Surgery, Sohar Hospital, Sohar Al-Batinah, OMN; 2 Department of Internal Medicine, Carepoint Health Bayonne Medical Center, New Jersey, USA; 3 Intensive Care Unit, Abbasi Shaheed Hospital, Karachi, PAK; 4 Department of Internal Medicine, Ameer-ud-Din Medical College/Lahore General Hospital, Lahore, PAK; 5 Department of Medicine, Combined Military Hospital (CMH), Rawalpindi, PAK; 6 Department of Hematology Oncology, Queens Hospital, London, GBR

**Keywords:** clinical outcomes, computed tomography, glasgow coma scale, intracranial pressure, mortality, optic nerve sheath diameter, recovery, traumatic brain injury, ultrasonography

## Abstract

Objective

This study aimed to determine the extent to which ultrasonographic and computed tomographic (CT) measurements of the optic nerve sheath diameter (ONSD) correlate with the prognosis and severity of traumatic brain injury (TBI).

Methodology

A prospective cohort study was conducted among 200 adult patients admitted to a Level 1 trauma center over one year. All patients were between 18 and 70 years of age and had sustained mild to severe TBI. Based on the Glasgow Coma Scale (GCS), patients were stratified into three categories of injury severity: mild, moderate, and severe. The ONSD was measured using both bedside ultrasonography and CT imaging. CT scans served as the reference standard for identifying intracranial pathologies. The primary objective was to evaluate whether ONSD measurement could help identify patients with intracranial hypertension (elevated intracranial pressure, ICP). CT findings, mortality, and long-term neurological outcomes were analyzed to assess the association between ONSD values and clinical prognosis.

Results

A moderate to strong positive correlation was found between the ONSD measured by ultrasonography and the severity of TBI, as assessed by the GCS. Increasing injury severity was associated with larger ONSD values. A significant association was also observed between ONSD measurements and CT indicators of elevated ICP, including midline shift, basal cistern effacement, and brain herniation. Moreover, higher ONSD values were significantly associated with poorer clinical outcomes, including increased mortality and delayed neurological recovery. The relationship between ONSD and these outcome measures appeared strong and consistent.

Conclusion

Ultrasound-based measurement of ONSD proved to be a reliable, non-invasive indicator of TBI severity and intracranial pressure. It demonstrated significant correlation not only with the degree of brain injury but also with patients’ clinical outcomes. Overall, these findings support the use of ONSD as a practical and effective surrogate tool for assessing raised ICP, particularly in situations where direct measurement is not feasible.

## Introduction

Traumatic brain injury (TBI) is one of the leading causes of death and disability worldwide. Early detection of secondary brain injuries, such as intracranial hypertension, is crucial for enhancing patient outcomes. Traditionally, intracranial pressure (ICP) monitoring in severely injured patients has been invasive, providing accurate real-time data on brain pressure and the patient’s clinical condition [1]. The Brain Trauma Foundation recommends invasive ICP monitoring as the reference standard for severe TBI. However, it carries procedural risks and is not available in all healthcare settings. The search for safer, non-invasive methods to monitor ICP at the bedside and through imaging has been driven by both clinical necessity and the goal of minimizing patient risk [2].

The optic nerve sheath is an elastic structure that is continuous with the subarachnoid space and therefore shares its characteristics. In cases of intracranial hypertension, the optic nerve sheath expands because the optic nerve itself cannot [3]. Understanding this physiological mechanism allows clinicians to relate the state of the optic nerve sheath to intracranial pressure variations and cerebrospinal fluid (CSF) dynamics, from normal circulation to elevated ICP. This relationship provides a valuable basis for evaluating changes in ICP using the optic nerve sheath as an indirect indicator [4].

Focused ultrasound assessment of the optic nerve sheath offers several advantages. It is rapid, portable, and repeatable. Unlike MRI or CT, it does not require a confined space or patient transfer. Point-of-care ultrasonography (POCUS) can be used to evaluate the optic nerve sheath diameter (ONSD) in the clinic, at the patient’s bedside, or even in prehospital environments. This provides clinicians with a non-invasive, readily accessible tool for assessing intracranial pressure [5].

Similarly, ONSD can be measured using computed tomography (CT) of the head. Studies comparing CT-derived ONSD with direct ICP measurements have shown a strong correlation between the two [5]. Interestingly, CT-measured ONSD may correlate even more closely with elevated ICP than traditional CT indicators such as midline shift, basal cistern effacement, or herniation, which are often subjective and variable in interpretation [6,7].

CT assessment of ONSD complements ultrasound when sonographic imaging is technically limited, although such situations are uncommon. Both modalities correlate well with increased ICP, but ultrasound remains the preferred technique at the bedside because of its accessibility and ease of repetition [8]. Currently, there are no universally accepted criteria for interpreting CT-derived ONSD in conjunction with clinical findings, as measurement techniques and threshold values vary among studies [9]. However, several reports suggest that an ONSD of 5.0 mm or greater should raise concern, since higher values are frequently seen in patients with elevated ICP [10,11].

The present study was designed to examine the relationship between ONSD, as measured by ultrasonography and CT, and the severity and outcomes of TBI. By assessing both modalities concurrently, this study aimed to clarify their complementary roles in early risk stratification and continuous monitoring of TBI patients. This work is significant because it may support the validation of ONSD as a practical indicator in clinical settings and help bridge the gap between bedside suspicion of elevated ICP and evidence-based management guided by TBI clinical practice guidelines.

## Materials and methods

Study design

This prospective observational study was conducted in the Emergency Department (ED) of a Level 1 trauma center at Karachi Medical and Dental College (KMDC). The study examined the relationship between emergency optic nerve sheath diameter (ONSD) measurements obtained by ultrasonography and the severity and outcomes of head injuries. The Glasgow Coma Scale (GCS) was used to assess the severity of the injury [12]. Outcomes were evaluated in terms of nonsurgical clinical recovery, mortality, and long-term neurological function. Severe intracranial pathology confirmed by computed tomography (CT) served as the gold standard in this study. The primary focus was on brain pathology leading to elevated intracranial pressure (ICP) and its physical manifestations, as related to the patient’s neurological status measured by the GCS.

Population and sample collection

A total of 200 adult patients, aged 18 to 70 years, who sustained mild, moderate, or severe traumatic brain injuries were enrolled. Severity was classified into three categories based on GCS scores: 13-15, 9-12, and ≤8 for mild, moderate, and severe TBI, respectively. All patients presented with clinical signs necessitating an immediate CT scan. Although there was overlap in clinical features across severities, these variations were consistent with known pathophysiological mechanisms of TBI.

Data collection

Both ultrasonography and CT imaging were used to measure the diameter of the optic nerve sheath (ONSD). These modalities were applied to evaluate the extent of head trauma and its relation to patient outcomes. For CT, a standardized protocol was followed: 350 mA and 120 kV at a 0.9 pitch over a 25 cm field of view, producing a 1 cm field of view that encompassed all relevant anatomy. Slice thickness was 5 mm (reconstructed at 3 mm), and images were analyzed using a Hounsfield window set at 35% of the total dynamic range (minimum 1250, maximum 520). These parameters ensured optimal visualization of the ONSD and adjacent soft tissue structures. Data collection and archiving procedures were standardized to maintain traceability for subsequent analysis.

Ultrasonography was performed using a high-frequency linear probe (7.5-13 MHz) in B-mode, with patients lying supine and eyelids gently closed to avoid pressure on the ocular globe. A thick layer of gel was applied to prevent mechanical distortion of ocular structures. The optic nerve sheath diameter (ONSD) was measured exactly 3 mm posterior to the globe within the anterior intraorbital segment, the most sensitive site for detecting sheath distension due to elevated intracranial pressure (ICP). This measurement site was used consistently for all patients. Transverse and longitudinal views were obtained for each eye, and the ONSD was measured from outer dural margin to outer dural margin perpendicular to the nerve axis. Measurements were taken for both eyes, and the mean value was used for analysis. The same anatomical site, fixed 3-mm depth, probe orientation, imaging parameters, and measurement technique were applied uniformly to all patients to ensure reproducibility. All ultrasonographic assessments were performed by physicians trained in ocular ultrasound, and images were archived for secondary review and quality assurance.

All participants or their legal guardians provided informed consent before inclusion. The study was conducted in accordance with institutional ethical standards and approved by the Institutional Review Board of Karachi Medical and Dental College (Approval No. KMDC/IRB/PG/2022/551).

Statistical analysis

Statistical analysis aimed to determine the correlation between ultrasonographic ONSD and CT evidence of elevated ICP in head-injury patients. Statistical analysis was conducted using IBM SPSS Statistics version 26.0 (IBM Corp., Armonk, USA). Continuous variables, including optic nerve sheath diameter (ONSD), were expressed as mean ± standard deviation (SD). Categorical variables such as gender, mechanism of injury, CT findings, and clinical outcomes were summarized as frequencies and percentages. The Pearson correlation coefficient (r) was used to evaluate the association between ultrasonographic and CT-derived ONSD measurements and indicators of traumatic brain injury severity, including Glasgow Coma Scale (GCS) scores, CT signs of raised intracranial pressure, mortality, clinical recovery, and long-term neurological outcomes. Group comparisons across injury severity categories (mild, moderate, severe) were performed using one-way analysis of variance (ANOVA) for continuous variables and the chi-square test for categorical variables. All statistical tests were two-tailed, and a p-value < 0.05 was considered statistically significant.

## Results

A total of 200 participants were included in the study. The age of the participants ranged from 18 to 70 years. Among them, 120 (60%) were males, and 80 (40%) were females. The majority of patients sustained blunt force injuries, accounting for 160 (80%) cases, while 40 (20%) had penetrating injuries. Based on the Glasgow Coma Scale (GCS), 120 (60%) patients had mild injuries, 50 (25%) had moderate injuries, and 30 (15%) had severe injuries (Table 1).

**Table 1:** Participant demographics and injury characteristics GCS: Glasgow Coma Scale [12]

Parameter	Value	n (%)
Total Participants	200	-
Age Range	18-70 years	-
Gender	Male	120 (60%)
	Female	80 (40%)
Mechanism of Injury	Blunt Force	160 (80%)
	Penetrating	40 (20%)
Injury Severity (GCS value range)	Mild (13–15)	120 (60%)
	Moderate (9–12)	50 (25%)
	Severe (≤8)	30 (15%)

The mean optic nerve sheath diameter (ONSD) increased proportionally with the severity of the injury. Patients with mild head injury (GCS 13-15) had a mean ONSD of 4.5 ± 0.5 mm, those with moderate injury (GCS 9-12) had a mean of 5.3 ± 0.6 mm, and patients with severe injury (GCS ≤ 8) showed the highest mean ONSD of 6.2 ± 0.7 mm (Table 2).

**Table 2:** Ultrasonographic findings showing mean optic nerve sheath diameter (ONSD) across different injury severity levels GCS: Glasgow coma scale [12]

Injury Severity	Total number of participants	Mean ONSD (mm)	Standard Deviation
Mild (GCS 13–15)	120	4.5	0.5
Moderate (GCS 9–12)	50	5.3	0.6
Severe (GCS ≤8)	30	6.2	0.7

Among the CT findings, midline shift was observed in 40 (20%) cases, basal cistern effacement in 50 (25%), herniation in 30 (15%), and ventricular compression in 35 (17.5%) cases. All findings showed a significant positive correlation with ultrasonographic ONSD (r = 0.58-0.70, p < 0.05) (Table 3).

**Table 3:** Correlation between ultrasonographic ONSD and CT findings (ICP indicators) *significant p-value (a p-value less than 0.05 was considered significant) ONSD: optic nerve sheath diameter; ICP: intracranial pressure

CT findings	Number of Cases (%)	Pearson (r)	t-statistics (from Pearson test	Statistical Significance
Midline Shift	40 (20%)	0.65	6.12	<0.001*
Basal Cistern Effacement	50 (25%)	0.62	5.78	<0.001*
Herniation	30 (15%)	0.70	6.85	<0.001*
Ventricular Compression	35 (17.5%)	0.58	5.22	<0.001*

Mortality was observed in two (1.67%) patients with mild TBI, eight (16%) patients with moderate TBI, and 10 (33.33%) patients with severe TBI. Clinical recovery was achieved in 118 (98.33%) patients with mild injuries, 42 (84%) with moderate injuries, and 10 (33.33%) with severe injuries. Preservation of long-term neurological function was noted in 115 (95.83%) patients with mild TBI, 35 (70%) with moderate TBI, and eight (26.67%) with severe TBI (Table 4).

**Table 4:** Outcome based on injury severity and ONSD measurements GCS: Glasgow coma scale [12]

Outcome Measure	Mild Injury (GCS 13–15)	Moderate Injury (GCS 9–12)	Severe Injury (GCS ≤8)
Mortality Rate n (%)	2 (1.67%)	8 (16%)	10 (33.33%)
Clinical Recovery n (%)	118 (98.33%)	42 (84%)	10 (33.33%)
Long-Term Neurological Function n (%)	115 (95.83%)	35 (70%)	8 (26.67%)

Both ultrasonographic and CT-derived ONSD measurements showed significant correlations with injury severity and outcomes. Ultrasonographic ONSD demonstrated a moderate positive correlation with GCS-based injury severity (r = 0.52, p < 0.01) and strong positive correlations with CT findings, including midline shift (r = 0.65), basal cistern effacement (r = 0.62), herniation (r = 0.70), and ventricular compression (r = 0.58), all p < 0.05. ONSD also correlated moderately with mortality (r = 0.45, p < 0.01) and showed negative correlations with clinical recovery (r = -0.39, p < 0.05) and long-term neurological function (r = -0.42, p < 0.05). Similarly, CT-derived ONSD showed a strong positive correlation with injury severity (r = 0.60, p < 0.01) and mortality (r = 0.49, p < 0.01), as well as a moderate negative correlation with clinical recovery (r = -0.44, p < 0.05) (Table 5).

**Table 5:** Correlation of ultrasonographic and CT-derived ONSD with injury severity and clinical outcomes A p-value less than 0.05 was considered significant. ONSD: optic nerve sheath diameter; TBI: traumatic brain injury; ICP: intracranial pressure; GCS: Glasgow Coma Scale

Analysis Factor	Pearson correl (r)	Statistical Significance (p-value)	Interpretation
Ultrasonographic ONSD vs. Injury Severity (GCS)	0.52	< 0.01	There was a moderate positive correlation between ultrasonographic ONSD and the severity of injury as measured by the GCS. Higher ONSD values were associated with more severe head injuries.
Ultrasonographic ONSD vs. Midline Shift (CT)	0.65	< 0.05	A strong positive correlation was found between ultrasonographic ONSD and midline shift on CT. Larger ONSD was associated with greater midline displacement, indicative of higher ICP.
Ultrasonographic ONSD vs. Basal Cistern Effacement (CT)	0.62	< 0.05	The correlation between ONSD and basal cistern effacement was significant, indicating that larger ONSD measurements were related to more severe effacement of basal cisterns, suggesting elevated ICP.
Ultrasonographic ONSD vs. Herniation (CT)	0.70	< 0.01	A very strong positive correlation was found between ONSD and herniation on CT. The larger the ONSD, the greater the likelihood of brain herniation, a serious sign of increased ICP.
Ultrasonographic ONSD vs. Ventricular Compression (CT)	0.58	< 0.05	A moderate positive correlation between ONSD and ventricular compression was observed, suggesting that larger ONSD values were linked to increased pressure leading to compression of the ventricles.
Ultrasonographic ONSD vs. Mortality Rate	0.45	< 0.01	A moderate correlation was found between ONSD and mortality. Larger ONSD was associated with a higher mortality rate in TBI patients, especially those with severe injuries.
Ultrasonographic ONSD vs. Clinical Recovery	-0.39	< 0.05	There was a moderate negative correlation between ONSD and clinical recovery, indicating that larger ONSD was associated with poorer clinical recovery outcomes.
Ultrasonographic ONSD vs. Long-Term Neurological Function	-0.42	< 0.05	A moderate negative correlation was found between ONSD and long-term neurological outcomes. Larger ONSD values predicted worse long-term neurological function, likely due to elevated ICP during injury.
CT ONSD vs. Injury Severity (GCS)	0.60	< 0.01	A strong positive correlation between CT-derived ONSD and GCS injury severity was found, with increased ONSD corresponding to more severe brain injury and lower GCS scores.
CT ONSD vs. Mortality Rate	0.49	< 0.01	A moderate positive correlation was observed between CT-derived ONSD and mortality. Higher ONSD values on CT were associated with higher mortality, especially in severe TBI cases.
CT ONSD vs. Clinical Recovery	-0.44	< 0.05	A moderate negative correlation between CT-derived ONSD and clinical recovery indicated that patients with higher ONSD on CT had lower chances of full recovery.

## Discussion

The study aimed to better understand how the optic nerve sheath diameter (ONSD) relates to the severity and clinical outcomes of traumatic brain injury (TBI). To achieve this, ultrasonography and computed tomography (CT) were used to directly measure the ONSD in a cohort of individuals with TBI. These measurements were then compared with established indices of TBI severity, such as the Glasgow Coma Scale (GCS), and with additional metrics assessing TBI outcomes, including the degree of intracranial pressure (ICP) elevation at the time of injury.

A significant and moderate association was found between ONSD and TBI severity as measured by the GCS. Among patients with low GCS scores (≤8), it was expected that a larger ONSD would be observed; the mean ONSD in this group was 6.2 mm. In contrast, patients with higher GCS scores (13-15) exhibited smaller, near-normal ONSD values (approximately 4.5 mm), indicating minimal or no intracranial hypertension. In a study by Munawar et al. [13] involving 10 TBI patients with GCS scores ranging from 4 to 15, ONSD measurements varied between 4.0 and 6.8 mm, with the lower range too close to normal to suggest serious brain injury.

The present study also investigated the relationship between ONSD and intracranial pressure (ICP) in patients with TBI. A strong positive correlation was observed between ONSD measured by ultrasound and CT findings suggestive of raised ICP, including midline shift, basal cistern effacement, and herniation. Larger ONSD values were associated with more severe CT abnormalities, reflecting higher ICP levels. These findings align with those of Robba et al., who demonstrated a significant correlation between ultrasonographic ONSD and imaging indicators of ICP, thereby supporting ONSD as a non-invasive diagnostic marker for elevated ICP in TBI patients [14].

The study further examined the relationship between ONSD and clinical outcomes, including mortality and long-term neurological recovery. Despite the observed correlations with imaging findings, ONSD showed limited predictive value for these clinical outcomes. Previous studies by Young et al. [15] and Wang et al. [16] reported that larger ONSD measurements were predictive of poorer outcomes, including prolonged neurological dysfunction and higher mortality rates. However, the present findings suggest that ONSD may not always reliably reflect the proper degree of intracranial hypertension or that the pathophysiological changes in TBI may alter the typical ICP-ONSD relationship.

A comparison between ultrasonographic and CT-based measurements of ONSD demonstrated that both modalities correlated well with brain injury severity and ICP-related changes. However, ultrasonography offered several practical advantages, including its bedside applicability, repeatability, and non-invasive nature, making it a more suitable tool for ongoing monitoring. These results are consistent with previous studies linking optic nerve and visual pathway edema, as assessed via ultrasound, to the severity of brain injury. Similarly, Lim et al. reported that ONSD measurement by ultrasonography is clinically valuable for detecting elevated ICP [9].

Limitations

Despite promising results, this study has several limitations. Intracranial pressure was not monitored directly, preventing a one-to-one comparison between ONSD and actual ICP measurements. While ultrasonography provides non-invasive ONSD values, these measurements do not directly quantify intracranial pressure. Future studies should incorporate invasive ICP monitoring alongside ONSD measurement to validate this correlation. Overall, the lack of direct comparison limits the extent to which ONSD can be interpreted as an accurate in vivo marker of human ICP.

## Conclusions

This study demonstrated that ultrasonographic measurement of the optic nerve sheath diameter (ONSD) is an effective method for assessing traumatic brain injury (TBI). The technique allows for detailed evaluation of several key aspects of TBI, including injury severity, changes in intracranial pressure, and even patient prognosis. Its reliability, real-time applicability, non-invasive nature, and ease of repetition make it a valuable bedside tool for continuous monitoring in patients with TBI.
